# Pedunculoside targets P2X7R to protect against myocarditis by regulating the NLRP3/PIP2/MAPK signaling pathway

**DOI:** 10.3389/fphar.2025.1589298

**Published:** 2025-06-26

**Authors:** Youqiong Zhuo, Jia He, Qin-Qin Wang, Yuntian Xiao, Xiaoyun Xie, Lina Liu, Shilin Yang, Jingjing Li, Renyikun Yuan, Hongwei Gao

**Affiliations:** ^1^ College of Pharmacy, Guangxi University of Chinese Medicine, Nanning, China; ^2^ School of Food Science and Technology, Nanchang University, Nanchang, China; ^3^ College of Pharmacy, Jiangxi University of Chinese Medicine, Nanchang, China; ^4^ Department of Rehabilitation Sciences, Faculty of Health and Social Sciences, Hong Kong Polytechnic University, Kowloon, Hong Kong SAR, China; ^5^ The Research Centre for Chinese Medicine Innovation, Hong Kong Polytechnic University, Kowloon, Hong Kong SAR, China

**Keywords:** pe, myocarditis, P2X7R/NLRP3/IL-1β, PIP2, MAPK

## Abstract

**Background:**

Myocarditis is an inflammation of the myocardium caused by a variety of reasons, with myocardial cell necrosis and interstitial inflammatory cell infiltration as the main manifestations. Pedunculoside (PE) plays a protective role in inflammatory diseases; however, it’s effect and mechanism on myocarditis remains unexplored.

**Methods:**

In this study, we evaluated the cardioprotective effects of PE *in vivo* and *in vitro* using the LPS + ATP-induced cardiomyocyte injury model and the LPS-induced rat myocarditis model, and elucidated its potential mechanism.

**Results:**

We found that PE demonstrated inhibition of H9c2 cell death and decreased ROS, Ca^2+^ levels, and MMP loss induced by LPS + ATP. Moreover, PE improved cardiac function in LPS-induced myocarditis rats. Mechanistically, PE suppressed the activation of the NLRP3 inflammasome, PIP2, and MAPK signaling pathways, which are associated with P2X7R. Additionally, PE interfered with and attenuated the interaction between P2X7R and PIP2, displaying strong docking activity with P2X7R.

**Conclusion:**

Taken together, PE exhibited significant anti-myocarditis activity by interacting with P2X7R and inhibiting the NLRP3, PIP2, and MAPK pathways, highlighting its potential as a therapeutic agent for clinical myocarditis treatment.

## 1 Introduction

Myocarditis, characterized by inflammatory lesions in the myocardium, arises from various factors such as infection, toxin exposure, and immune activation (Han et al., 2023). Pathogenic mechanisms, including calcium dysregulation, inflammation, oxidative stress, and mitochondrial dysfunction, contribute to organ dysfunction (Kawakami et al., 2021). Bacterial infections and toxins can cause severe myocardial damage (Wang et al., 2021). Clinically, mild patients have no obvious symptoms, while severe patients may experience heart failure, arrhythmia and even sudden death (Nie et al., 2022). Currently, there is still no specific treatment for myocarditis, but studies have reported that Chinese herbal medicine has a good effect on improving myocarditis (Chen et al., 2020). Therefore, there is a critical need to identify effective compounds of Chinese herbal medicine for the treatment of myocarditis.

P2X7R, a bifunctional membrane ion channel, regulates various physiological functions, including eliminating infectious organisms, regulating inflammatory processes, and inducing cell death (Xu et al., 2020). P2X7R plays a critical role in multiple aspects of cardiovascular diseases. Studies have demonstrated that P2X7R knockout significantly alleviated angiotensin II (Ang II)-induced myocardial injury (Zhong et al., 2024). In diabetes-related heart injury, blocking P2X7R improved heart problems through PKCβ and ERK pathway (Huang et al., 2021). Additionally, P2X7R antagonists effectively attenuated pathological injury in experimental autoimmune myocarditis by suppressing immune cell activity as well as downregulating the expression of NADPH oxidase 2/4 (Zempo et al., 2015). What’s more, P2X7R activation initiates the inflammasome pathway, leading to the release of mature IL-1β and aiding in the elimination of infectious organisms such as *Toxoplasma gondii*, *Staphylococcus aureus*, and *Escherichia coli* (Bhattacharya and Jones, 2018). The NLRP3 inflammasome, a crucial cytoplasmic multi-protein complex, senses and responds to infections caused by diverse pathogens, playing a significant role in host defense mechanisms (Han et al., 2022). Phosphatidylinositol-4,5-bisphosphate (PIP2), a crucial signaling molecule, regulates various cellular functions, including cell membrane dynamics, cell morphology, differentiation, proliferation, and ion transport. Upon activation, PLC hydrolyzes PIP2 to diacylglycerol (DAG) and inositol 1, 4, 5-trisphosphate (IP3), acting as second messenger in cell signaling (Lowe et al., 2022). IP3 facilitates intracellular Ca^2+^ release, while PIP2 also severs as an epigenetic regulator of ribosomal RNA gene transcription (Yang et al., 2021). Therefore, PIP2 may modulate inflammation by inhibiting the activation of NLRP3 inflammasome. Furthermore, MAPK signaling pathway serves as a conduit for numerous inflammatory factors, regulating cellular processes like proliferation, differentiation, and apoptosis (Chen et al., 2023). Inhibition of the MAPK signaling pathway has been demonstrated to reduce inflammatory factor release, suggesting its potential as a therapeutic target (Zhao et al., 2021). Hence, modulation of the P2X7R/NLRP3/IL-1β, PIP2 and MAPK signaling pathways could offer an effective approach for treating inflammatory diseases.

Jiubiying, derived from the dry bark of *Ilex rotunda* Thunb., exhibits antibacterial analgesic, anti-inflammatory, and anti-tumor effects (Chen et al., 2019). Previous studies of Jiubiying primarily focused on its efficacy in treating gastrointestinal and infectious diseases, and recent findings suggest that Jiubiying has a notable cardiovascular protective effect (Li et al., 2021). Given this potential, developing Jiubiying into an effective treatment for cardiovascular diseases holds promising prospects. PE, the main active compound isolated from Jiubiying, is an ursane type pentacyclic triterpenoid saponin. *Chinese Pharmacopoeia* 2020 stipulates PE content in Jiubing dry products to be not less than 4.5%, serving as a quality indicator. PE exhibits diverse pharmacological activities, including anti-tumor, anti-inflammatory, and cardiovascular protective effects (Zeng et al., 2022). However, its potential to protect against myocarditis and the underlying mechanism remains largely unknown. Building on the pathogenic role of P2X7R in myocarditis and the cardiovascular protective properties of PE, we hypothesized that PE may play a protective role against myocarditis by inhibiting P2X7R and regulate the P2X7R/NLRP3/IL-1β, PIP2 and MAPK signaling pathways, thereby alleviating the inflammatory infiltration and damage of myocardial tissue. Investigating the cardiovascular protective targets and mechanisms of PE both *in vitro* and *in vivo* may provide a theoretical basis for the development of novel anti-cardiovascular disease drugs.

## 2 Materials and methods

### 2.1 Chemical and reagents

PE was isolated from Jiubiying in our laboratory with a purity greater than 98%, which was tested by HPLC. LPS, LDH, and BCA protein detection kits were obtained from Beyotime (Shanghai, China). ATP was purchased from Macklin (Shanghai, China). (4, 5-Dimethylthiazol-2-yl)-2, 5-diphenyltetrazolium bromide (MTT), 2′, 7′-Dichlorodihydrofluorescein diacetate (DCFH_2_-DA), Fluo-3a.m., Hoechst 33,342 and ethylene glycol tetra acetic acid (EGTA) were purchased from Sigma-Aldrich (St. Louis, MO, United States). PE-Annexin V Apoptosis Detection Kit was bought from BD PharmingenTM (Becton-Dickinson, NJ, United States). From Thermo Fisher Scientific (Waltham, MA, United States), Mitochondrial membrane potential assay kit (JC-1), PierceTM Protein A/G Magmetic Beads and BCA protein detection kit were obtained. Dulbecco’s modifiedeagle medium (DMEM) and fetal bovine serum (FBS) were acquired from Gibco (Grand Island, NY, United States). AZD9056 was acquired from MedChemExpress (New Jersey, United States). IL-1β and TNF-α ELISA kits were obtained from Neobioscience (Shenzhen, China). SOD, MDA, ALT and AST detection kits were purchased on selection from Nanjing Jiancheng Bioengineering Institute (Nanjing, China), CK-MB detection kit bought from Shanghai Enzyme Linked Biotechnology Co. LTD. (Shanghai, China).

Antibodies for P2X7R (#13809), Cleaved-IL-1β (#63124), IL-1β (#31202) Cleaved Caspase-1 (#89332), PLCγ2 (#3872), IP3 Receptor1 (#8568), DAG Lipase α (#13626), p-JNK (#9255), JNK (#9252), p-ERK (#4370), ERK (#4695), p-p38 (#4511), p38 (#8690), GAPDH (#5174) and the secondary antibodies including anti-rabbit IgG, HRP-linked Antibody (#7074) as well as anti-mouse IgG, HRP-linked Antibody (#7076) were obtained from Cell Signaling Technology (Beverly, MA, United States). NLRP3 (DF7438), and ASC (DF6304) were purchased from Affinity Biosciences (Cincinnati, OH, United States), Caspase-1 (ab1872) were obtained from Abcam (Cambridge, MA, United States). PIP2 (#53412) were obtained from Senta (Santa Cruz Biotechnology, United States).

### 2.2 Cell culture

H9C_2_ cells were purchased from the Cell Bank of the Chinese Academy of Sciences (Shanghai, China) and cultured in DMEM with 10% FBS and 1% Penicillin/Streptomycin, incubated at 37°C in a humidified atmosphere with 5% CO_2_.

### 2.3 Cell viability assay

H9c2 cells were seeded into a 96-well plate at the density of 4 × 10^3^/well overnight. This was followed by exposure to a series of concentrations of PE (5, 10, 20, 40, 80 μM) or AZD9056 (2.5, 5, 10, 20, 40 μM) for 24 h, or pre-administered with PE (5, 10, 20 μM) for 4 h or AZD9056 (2.5, 5, 10, 20, 40 μM) for 1 h followed by incubated with LPS (1 μg/mL) for 12 h and subsequently ATP (10 mM) for another 24 h. After incubation, the absorbance was measured using a microplate reader (SYNERGYH1, Bio Tek, United States) by measuring an absorbance of 490 nm.

### 2.4 Measurement of lactate dehydrogenase (LDH)

H9c2 cells were seeded into a 96-well plate at the density of 4 × 10^3^/well overnight. This was followed by pre-administered with PE (5, 10, 20 μM) for 4 h, then acted with LPS (1 μg/mL) for 12 h and ATP (10 mM) for another 24 h. After which the culture supernatant was collected to detect LDH release, and the absorbance was measured at 450 nm using a multimode plate reader by measuring absorbance of 450 nm.

### 2.5 Apoptosis assay

H9c2 cells were seeded into a 96-well plate at the density of 4 × 10^3^/well overnight. This was followed by pre-administered with PE (5, 10, 20 μM) for 4 h, then acted with LPS (1 μg/mL) for 12 h and ATP (10 mM) for another 24 h. The cells were labeled with PI (1 μg/mL) and Hochest 33,342 (1 μg/mL), and then washed twice and recorded by fluorescence microscope (Leica, Wetzlar, Germany).

### 2.6 Flow cytometry

H9c2 cells were seeded into a 6-well plate at the density of 1.0 × 10^5^/well overnight. Cells were pre-administered with PE (20 μM) for 4 h followed by LPS (1 μg/mL) induction for 12 h and ATP (10 mM) induction for another 24 h. 400 μL of Binding Buffer (1×) was resuspended and then transferred into flow tubes; 3 μL of Annexin V and 7-AAD were added and mixed thoroughly, then incubated for 15 min; the cells were detected and analyzed by flow cytometry.

H9c2 cells were seeded into a 12-well plate at the density of 5.0 × 10^4^/well overnight. After this cells were treated with LPS (1 μg/mL) for 12 h and ATP (10 mM) for a series of times, or treated with PE (5, 10, 20 μM) for 4 h, followed by incubation with LPS (1 μg/mL, 12 h) and ATP (10 mM, 0.25 h or 10 h). The cells were labeled with ROS probe (DCFH_2_-DA 1 μM, 0.5 h) or Ca^2+^ probe (Fluo-3a.m. 1 μM, 1 h) respectively and then washed and collected for detection by the FACSMelodyTM Cell Sorter (BD bioscience, United States).

### 2.7 Fluorescence assay

H9c2 cells were seeded into a 96-well plate at the density of 4.0 × 10^3^/well overnight. After which cells were pre-administered with PE (5, 10, 20 μM) or AZD9056 (5 μM) for 4 h ahead of time and then induced by LPS (1 μg/mL) for 12 h and ATP (10 mM) for another 0.25 h, and then labeled with ROS probe (DCFH_2_-DA 1 μM, 30 min), MMP probe (10 μg/mL JC-1, 100 μL/well, 30 min) or PI dye (10 μg/mL, 10 min) and Hoechst33342 (1 μg/mL, 5 min). The cells were washed twice with PBS (100 μL/well). Ultimately, the cells were photographed and analyzed with a fluorescence microscope.

### 2.8 Immunofluorescence

As previously mentioned, the immunofluorescence of PIP2, NLRP3, Caspase-1, and ASC was investigated. Briefly, H9c2 cells were seeded into a confocal dish at the density of 8 × 10^4^/dish overnight (SPL, Pocheon, Korea). The cells were pre-treated with PE (20 μM) for 4 h or AZD9056 (5 μM) for 1 h before being stimulated with LPS (1 μg/mL) for 12 h and ATP (10 mM) for another 24 h. Cells were incubated with the corresponding primary antibody overnight at 4°C, followed by incubation with goat anti-mouse IgG Fluor^®^ 488 Conjugate (#S0017, 1:200) for 1 h at room temperature. After that, the cells were fixed and stained for 30 min with Hoechst 33,342 (1 mM) and were imaged with a confocal laser microscope (Leica, Wetzlar, Germany).

The cells were transfected with Plasmids of EGFP-NLRP3 for 36 h by using turboFect transfection reagents (#R0531, Thermo Fisher Scientific, Grand Island, NY, United States), followed by treatment with PE (40 μM) for 4 h and LPS (1 μg/mL) for 12 h and ATP (10 mM) for another 24 h, and the next steps were described above.

### 2.9 Western blot analysis

The study employed RIPA (1% PMSF and 1% cocktail) to extract total proteins of cells as well as the heart tissue. Total protein concentration was determined using the bicinchoninic acid (BCA) method (Thermo Fisher Scientific, United States). The denatured protein (20 μg in cell samples or 30 μg in animal tissue samples) was separated through 10% or 12% SDS-PAGE gel electrophoresis and electroblotting on PVDF membrane (Millipore, Billerica, MA, United States). After blocked with 5% skim milk up to 2 h at RT, the membrane was incubated with primary antibodies at 4°C overnight. The antibodies used in the study were as follows: anti-P2X7R (1:1000), NLRP3 (1:1000, ASC (1:1000), Cleaved-Caspase-1 (1:1000), Caspase-1 (1:1000), Cleaved-IL-1β (1:1000), IL-1β (1:1000), PIP2 (1:1000), PLCγ2 (1:1000), DAG (1:1000), IP3 (1:1000), p-JNK (1:1000, #4668), JNK (1:1000), p-ERK (1:1000), ERK (1:1000), p-p38 (1:1000) and p38 (1:1000). After that, the membrane was exposed to ChemiDoc™ MP Imaging System (Bio-Rad, Hercules, CA, United States) after incubating with secondary antibody including anti-rabbit IgG, HRP-linked Antibody (1:1000) and anti-mouse IgG, HRP-linked Antibody (1:1000).

### 2.10 Co-immunoprecipitation assay

Antigen samples were mixed with specific antibody (anti-PIP2) overnight at 4°C, and then protein A/G magnetic beads were added for 1 h. Immunoprecipitation products were eluted with SDS-PAGE reduction sample buffer.

### 2.11 Molecular docking

The structure of Peduncloside was downloaded from the PubChem database (https://pubchem.ncbi.nlm.nih.gov/) and converted to mol2 format for preservation. The structure of P2X7R was predicted by alpha fold in Uniport (https://www.uniprot.org) (ID = AF-Q99572-F1), download the complex crystal structure containing the original ligand, performed point charges on the target protein, and save it as a PDBQT format file. AutoDock4.2.6 software was used for molecular docking, and the binding energy (binding energy) ≤−5.0 kJ mol^−1^ was used as the screening basis. After which, Pymol 2.1 software was used to visualize the molecular docking results.

### 2.12 Cellular thermal shift assay (CETSA)

The HEK293T cells were lysed with RIPA Lysis Buffer (1% PMSF and 1% cocktail) and the cell lysates were co-incubated with vehicle control (DMSO) or PE (20 μM) for 0.5 h on ice, then centrifuged at 15,000 rpm for 20 min at 4°C. After which, the supernatant was divided into 6 parts on average and heated respectively at different temperatures (44, 47, 50, 53, 56, 59, 62, 65, 68°C and 71°C) for 3 min followed by cooling for 30 s at room temperature. Then the samples were centrifuged and the change of P2X7R protein level followed by temperatures after drug treatment was detected by Western blot assay.

### 2.13 Animals and ethical statement

All animal experimentations were approved by the Laboratory Animal Management Ethics Committee of Guangxi University of Chinese Medicine (Approval No. DW20230830-174). All experiments were in accordance with the Guidelines for the Care and Use of Laboratory Animals issued by the National Institutes of Health. The experimental animal production license numbers were SYXK (GUI-2019-0001). According to “Guangxi University of Traditional Chinese Medicine Care and Use of Laboratory Animals”, all animals received humane care. Healthy male SD rats (220–250 g) were purchased from Hunan Slack Jingda Laboratory Animal Co., Ltd. and acclimated for 1 week under standard SPF (specific pathogen-free environment) conditions. The rats were fed in SPF-grade rat cages at a room temperature of 25°C ± 5°C and relative humidity controlled at about 50%. Rat litter, rat chow, and drinking water were all sterile-sterilized.

### 2.14 Animal feeding, modeling, and administration

50 Male SD rats were randomly divided into 5 groups (n = 10): control group, LPS group, PE low-dose group (PE-L, 10 mg/kg), PE medium-dose group (PE-M, 20 mg/kg), and PE high-dose group (PE-H, 40 mg/kg). The PE administration group was given continuous tail vein administration for 5 days, once a day, while the rats in the control group and the LPS group were injected with physiological saline through the tail vein at the same time, and the model group and the administration group were then injected with LPS (8 mg/kg) intraperitoneally for 18 h (PE used in animals is made by the inclusion of hydroxypropyl-β-cyclodextrin, and the inclusion rate is 80%).

### 2.15 Detection of rat electrocardiogram and echocardiogram

After 18 h of intraperitoneal injection of LPS (8 mg/kg), the rats were anesthetized with 4 mL/kg chloral hydrate (10%). The ECG changes of the rats in each group were recorded in real-time, and MyLab™Six intelligent color ultrasonic diagnostic instrument (Maastricht, Netherlands) was used to record the Echocardiographic changes of rats in each group.

### 2.16 Routine analysis of blood

Rats were treated with LPS for the sake of inducing myocarditis. Blood was collected from anesthetized rats via the abdominal aorta. The amounts of WBC and Neu in blood were detected by Mindray hematology analyzer.

### 2.17 Determination of TNF-α, IL-6, SOD, MDA, CK-MB, ALT, and AST

With a tissue grinder (Tianjin, China), SD rats’ heart tissue was homogenized. The samples were centrifuged. Their supernatant was collected immediately and stored at −80°C. As per the manufacturer’s instructions, TNF-α and IL-6 levels were detected by ELISA kits, the SOD, MDA CK-MB, ALT, and AST were examined by corresponding kits.

### 2.18 Hematoxylin and eosin (H&E) staining

After the SD rats were treated with LPS for 18 h, the rats were anesthetized, the heart, and tissues were isolated, and fixed in 4% paraformaldehyde buffer for H&E staining.

### 2.19 Statistical analysis

All data were processed by GraphPad Prism 6.0 software (GraphPad Prism; San Diego, CA, United States), and all experiments were repeated at least three times. The results were presented as average value ±standard deviation (SD). The significance of differences between groups was determined by a one-way analysis of variance, after which Dunnett’s multiple comparisons test was used for *post hoc* analysis. When *P* < 0.05, results were considered to be statistically significant.

## 3 Results

### 3.1 PE exerted a protective effect against LPS + ATP-induced damage in H9c2 cells

**FIGURE 1:**
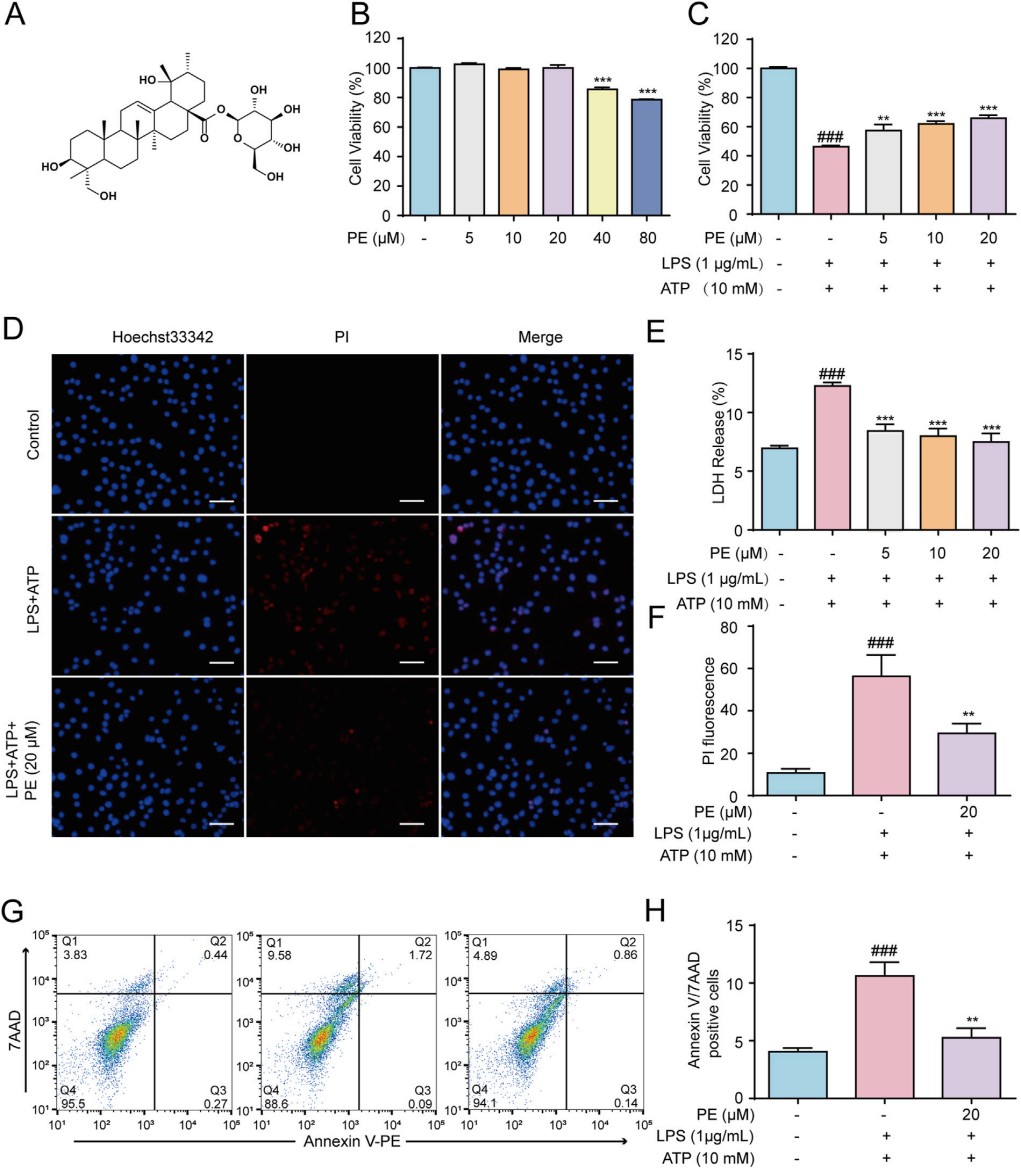
PE exerted a protective effect against LPS + ATP-induced damage in H9c2 cells. **(A)** The chemical structure of PE. **(B)** H9c2 cells were treated with PE (5, 10, 20, 40, 80 μM) for 24 h, the cytotoxicity of PE was detected by MTT assay. **(C)** H9c2 cells were treated with different concentrations of PE (5, 10, 20 μM) for 4 h, induced by LPS (1 μg/mL) for 12 h and ATP (10 mM) for 24 h. MTT assay was used to analyze the effect of PE on the survival rate of H9c2 cells induced by LPS + ATP (*n* = 3); **(D)** H9c2 cells were stained with Hoechst 33,342 and PI for 15 min. Images were obtained under fluorescence microscope (scale bar = 10 μm); **(E)** LDH assay was used to analyze the effect of PE on LDH release level of H9c2 cells induced by LPS + ATP (*n* = 3); **(F)** PI fluorescence statistics (*n* = 3); **(G,H)** H9c2 cells were stained with Annexin V/7-AAD and analyzed by flow cytometry (*n* = 3). Compared with control group, ^###^ *P* < 0.001; Compared with LPS + ATP group, ^**^ *P* < 0.01, ^***^ *P* < 0.001.

To preliminarily evaluate whether PE (Figure 1A) has a certain protective effect on LPS + ATP-induced H9c2 cell damage, the cell viability was firstly measured. The results showed that PE (5, 10, 20 μM) protected H9c2 cells from LPS + ATP damage to a certain extent without obvious cytotoxicity (Figures 1B,C). The result in Figure 1E hinted that PE (5, 10, 20 μM) significantly reduced the LDH release level of H9c2 cells induced by LPS + ATP. Simultaneously, the PI fluorescence and Annexin/7AAD staining results indicated that PE remarkably decreased LPS + ATP-induced cell death (Figures 1D,F–H). All in all, these results demonstrated that PE exerted striking protective effects on LPS + ATP-damaged H9c2 cells.

### 3.2 PE attenuated LPS + ATP-induced ROS generation and MMP loss in H9c2 cells

**FIGURE 2:**
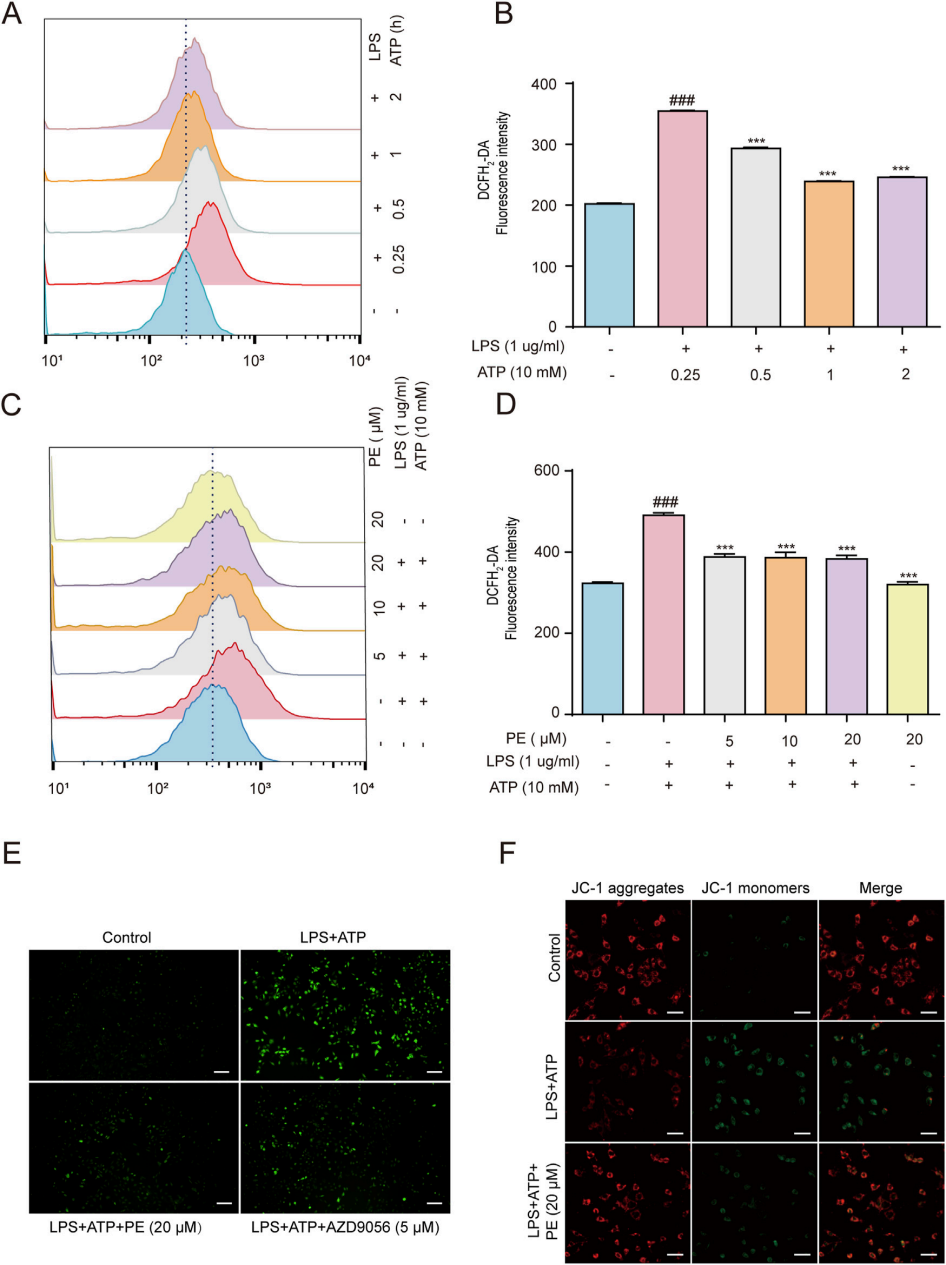
PE attenuated LPS + ATP-induced ROS generation and MMP loss in H9c2 cells. **(A,B)** H9c2 cells were induced by LPS (1 μg/mL) for 12 h and ATP (10 mM) for 0.25, 0.5, 1, 2 h. The cells were labeled with DCFH_2_-DA (1 μM) for 0.5 h, and the ROS levels were analyzed by flow cytometry (*n* = 3). **(C,D)** H9c2 cells were treated with different concentrations of PE (5, 10, 20 μM) for 4 h H9c2 cells were induced by LPS (1 μg/mL) for 12 h and ATP (10 mM) for 0.25 h. The cells were labeled with DCFH_2_-DA (1 μM) for 0.5 h, and the ROS levels were analyzed by flow cytometry (*n* = 3); **(E)** The images were obtained under fluorescence microscope. **(F)** H9c2 cells were pre-treated with PE (20 μM) for 4 h, LPS (1 ug/mL) for 12 h, ATP (10 mM) for 10 h, and JC-1 staining for 1 h. Images were obtained under fluorescence microscope) (scale bar = 10 μm). Compared with control group, ^###^ *P* < 0.001; Compared with LPS + ATP group, ^***^ *P* < 0.001.

Cell damage induced by LPS + ATP leads to the destruction of MMP and the generation of ROS(Song et al., 2022). The effect of PE on the level of cellular ROS and MMP was detected. The results in Figures 2A,B showed that LPS + ATP stimulated a sharp increase in ROS levels at 0.25 h compared with the control group. Based on which, flow cytometry and immunofluorescence results displayed that PE reduced the level of ROS (Figures 2C–E) and P2X7R inhibitor AZD9056 showed a similar effect. Taken together, results demonstrated that PE had a certain inhibitory effect on LPS + ATP-induced ROS levels in H9c2 cells, thus verifying that PE had a certain antioxidant activity. Mitochondrial membrane potential reflects the integrity of mitochondrial function and is a sensitive indicator for evaluating mitochondrial function. When the stability of MMP is abnormal, the normal physiological function of cells will be destroyed (Yang et al., 2025). MMP decreased after H9c2 cells were treated with LPS + ATP, in this case, the green fluorescence was significantly enhanced, whereas the red fluorescence intensity was greatly weakened. This was distinctly reversed when cells were treated with PE (20 μM). Overall, PE (20 μM) ameliorated LPS + ATP-stimulated MMP loss (Figure 2F).

### 3.3 PE inhibited the activation of NLRP3 inflammasome

**FIGURE 3:**
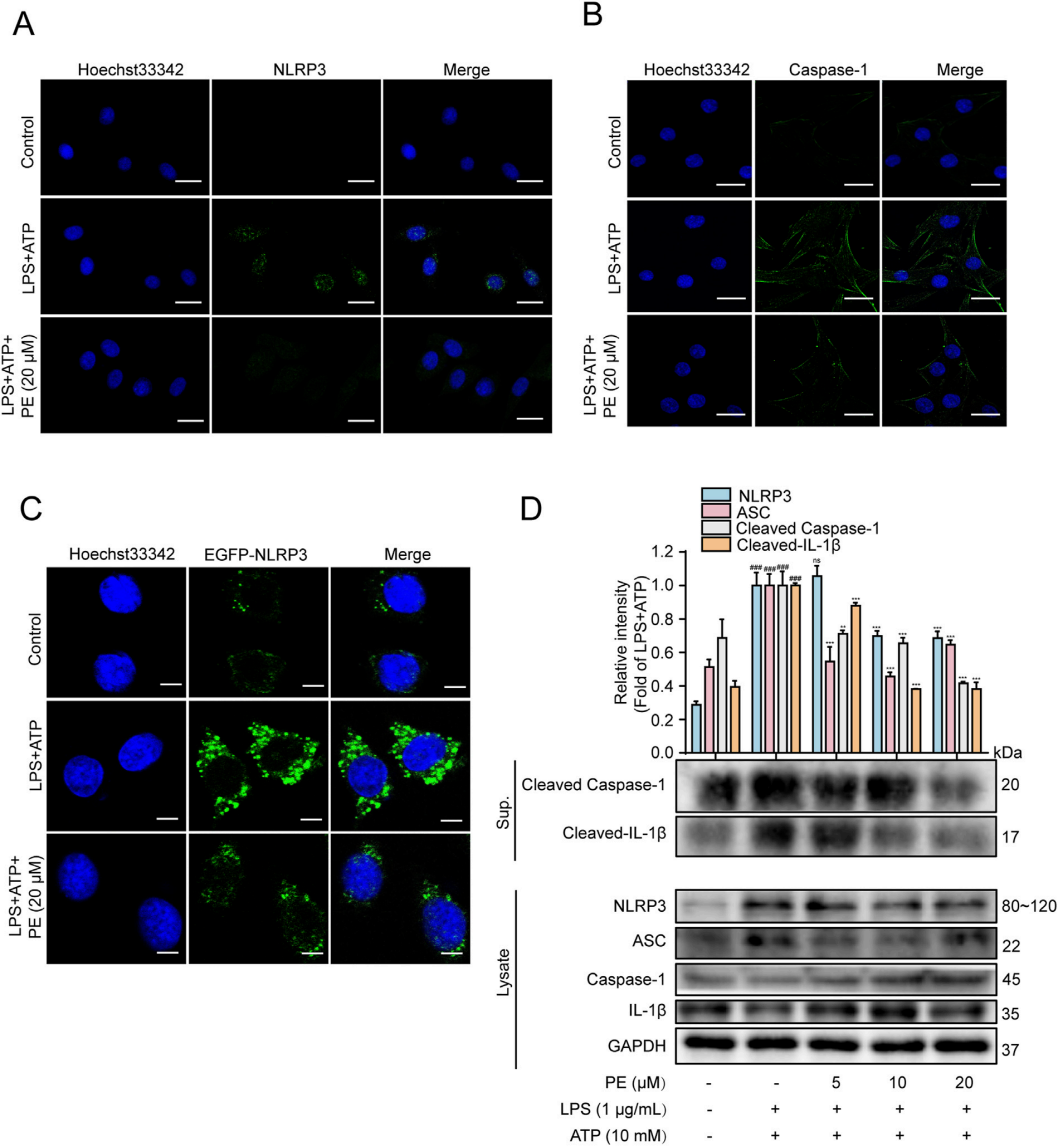
PE inhibited the activation of NLRP3 inflammasome. **(A)** The expression of NLRP3 in H9c2 cells was detected by immunofluorescence assay; **(B)** The activation of Caspase-1 in H9c2 cells was detected by immunofluorescence assay; **(C)** The expression of EGFP-NLRP3 in H9c2 cells was detected by immunofluorescence assay; **(D)** The protein expressions of NLRP3, ASC, Caspase1, Cleaved Caspase-1, IL-1β and Cleaved-IL-1β in H9c2 cells were detected by Western blot; scale bar = 20 μm (*n* = 3). Compared with control group, ^###^ *P* < 0.001; Compared with LPS + ATP group, ^**^ *P* < 0.01, ^***^ *P* < 0.001.

NLRP3 inflammasome is essential for host immune defense against bacterial, fungal, and viral infections (Wang et al., 2022). Stimulated by viral RNA, pore-forming toxins and ATP, NLRP3, Caspase-1 and ASC will form NLRP3 inflammasome, and activated Caspase-1 will further cut cytokine pro-IL-1β, and pro-IL-1β will be released outside the cell, triggering a series of inflammatory reactions in the body (Li et al., 2022). After activation of inflammasome, ASC will form ASC spots, which are supramolecular aggregates of ASC dimer and one of the means of Caspase-1 activation (Xia et al., 2021). Figures 3A,B fluorescence images showed that when H9c2 cells were stimulated by LPS + ATP, green fluorescence (NLRP3 fluorescence as well as Caspase-1 fluorescence) was heightened. Nevertheless, the above results were curbed after PE (20 μM) pretreatment. In addition, EGFP-NLRP3 was transfected into H9c2 cells to construct an NLRP3 overexpression model. As shown in Figure 3C, compared with LPS + ATP group, PE pretreatment reduced the green fluorescence of NLRP3 in H9c2 cells stimulated by LPS + ATP obviously, pointing out that PE inhibited NLRP3 expression. The Western blot data in Figure 3D indicated that when H9c2 cells were stimulated by LPS + ATP, the protein expressions of NLRP3, ASC, Cleaved Caspase-1, Cleaved-IL-1β increased observably, compared with the control group. The above protein expression was downregulated after pretreatment with different concentrations of PE (Figure 3D). In conclusion, PE inhibited the activation of NLRP3 inflammasome.

### 3.4 P2X7R facilitated LPS + ATP-induced cardiomyocyte injury

**FIGURE 4:**
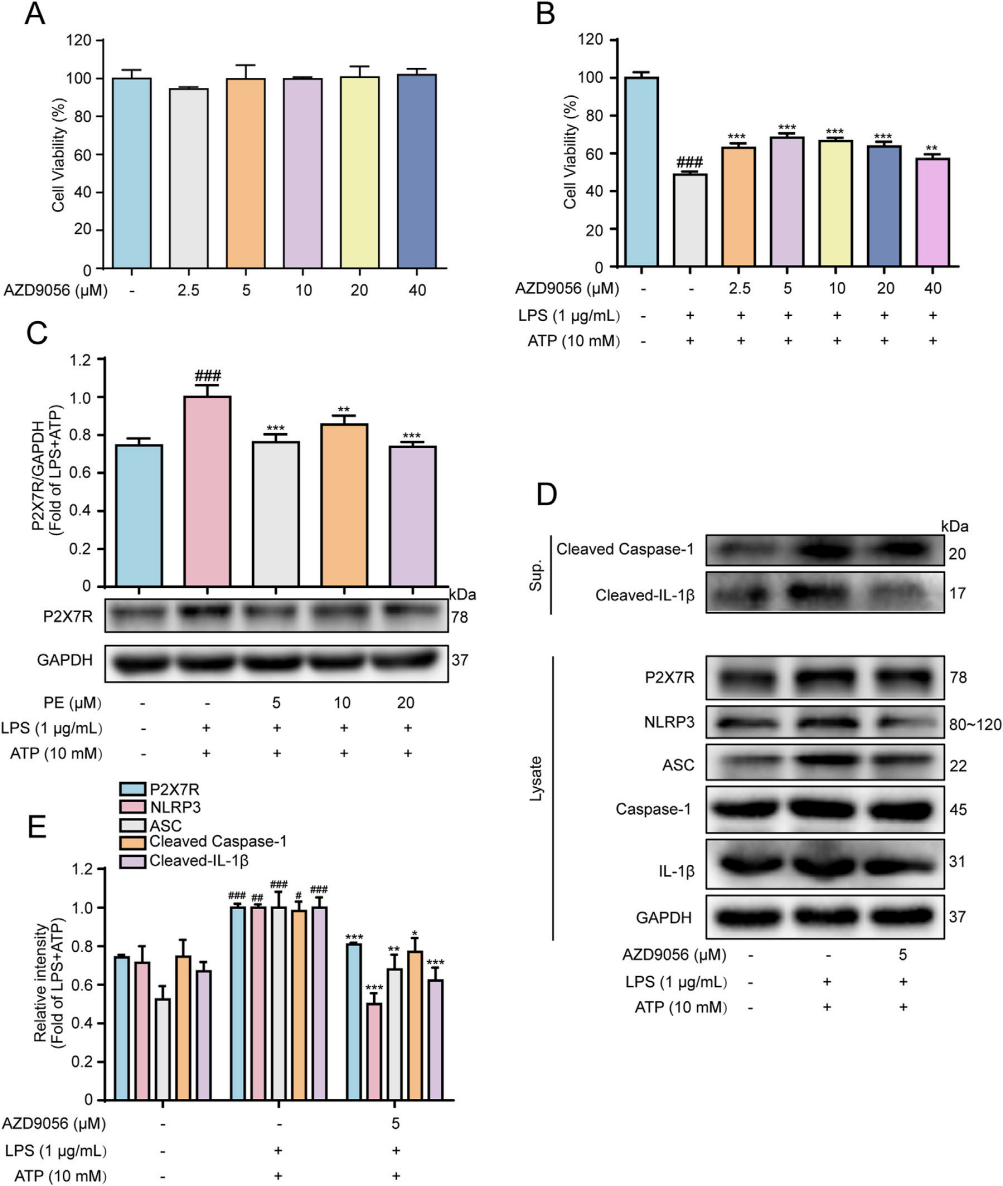
P2X7R facilitated LPS + ATP-induced cardiomyocyte injury. H9c2 cells were treated with AZD9056 (2.5, 5, 10, 20, 40 μM) for 24 h, and cell proliferation was detected by MTT assay. **(A)** The effect of AZD9056 on H9c2 cell viability; H9c2 cells were treated with AZD9056 (2.5, 5, 10, 20, 40 μM) for 1 h H9c2, then induced by LPS (1 μg/mL) for 12 h and ATP (10 mM) for 24 h (*n* = 3); **(B)** The effect of AZD9056 on the survival rate of H9c2 cells induced by LPS + ATP was determined by MTT assay (*n* = 3); **(C–E)** The protein expressions of P2X7R, NLRP3, ASC, Caspase-1, Cleaved Caspase-1, IL-1β and Cleaved-IL-1β in H9c2 cells were detected by Western blot (*n* = 3). Compared with control group, ^#^ *P* < 0.05, ^##^ *P* < 0.01, ^###^ *P* < 0.001; Compared with LPS + ATP group, ^*^ *P* < 0.05, ^**^ *P* < 0.01, ^***^ *P* < 0.001.

ATP can activate the P2X7R ion channel, and its expression is enhanced when the body is in an inflammatory state (Karmakar et al., 2016). NLRP3 is the convergence point of multiple IL-1β release signals, some of which may be related to P2X7 activation (Li et al., 2018). To further verify the effect of P2X7R on NLRP3-dependent IL-1β secretion, the P2X7R inhibitor AZD9056 was afforded for the following experiments. Figures 4A,B showed that, without toxicity to H9c2 cells, AZD9056 (2.5–40 μM) improved cell viability compared with LPS + ATP group, and AZD9056 (5 μM) worked better. Western blot data revealed that LPS + ATP induced a hike in the level of P2X7R compared with the control group, while PE (5, 10, 20 μM) pretreatment reduced the level of P2X7R signally (Figure 4C). Additionally, compared with the LPS + ATP group, the protein expressions of P2X7, NLRP3, ASC, Cleaved Caspase-1, and Cleaved-IL-1β were distinctly reduced after pretreatment with AZD9056 (5 μM) (Figures 4D,E). Taken together, P2X7R mediated NLRP3-dependent IL-1β secretion and promoted LPS + ATP-induced cardiomyocyte injury.

### 3.5 P2X7R activated PIP2 signaling pathway in LPS + ATP-induced H9c2 cells

**FIGURE 5:**
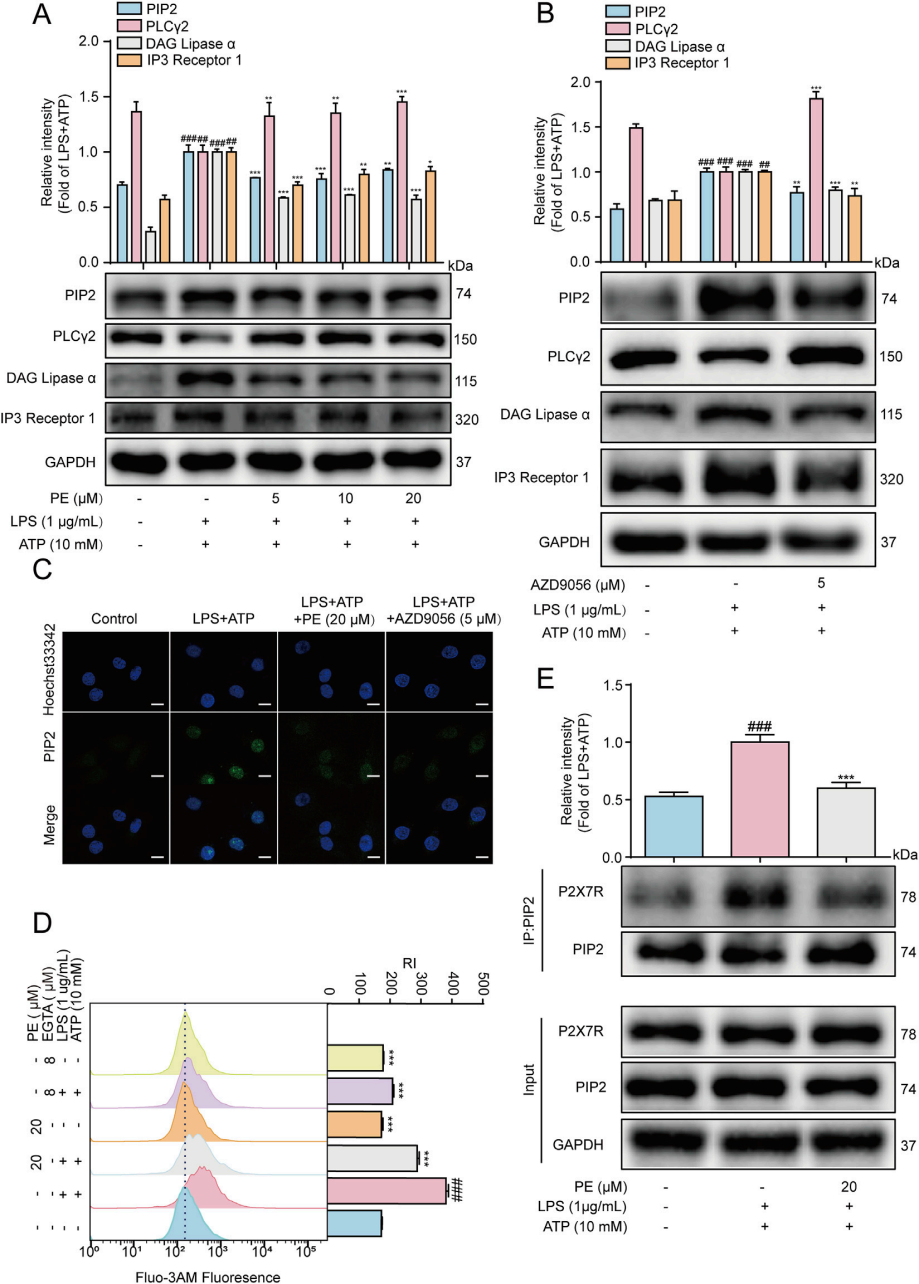
P2X7R activated PIP2 signaling pathway in LPS + ATP-induced H9c2 cells. H9c2 cells were treated with PE for 4 h or AZD9056 (5 μM) for 1 h, and then induced by LPS (1 μg/mL) for 12 h, ATP (10 mM) for 24 h or 10 h **(A,B)** The protein expression of P2X7R, PIP2, DAG, IP3 and PLCγ2 in H9c2 cells were detected by Western blot (*n* = 3); **(C)** The expression of PIP2 in H9c2 cells was detected by immunofluorescence assay (scale bar = 20 μm); **(D)** The cells were labeled with Fluo-3 a.m. (1 μM) for 1 h, and the Ca^2+^ level was detected by flow cytometry (*n* = 3); **(E)** The collected proteins were immunoprecipitated with PIP2 using magnetic beads, and immunocomplexes were determined by Western blot (*n* = 3). Compared with control group, ^##^ *P* < 0.01, ^###^ *P* < 0.001; Compared with LPS + ATP group, ^**^ *P* < 0.01, ^***^ *P* < 0.001.

As shown in Figure 5A, the results of Western blot showed that in the inflammatory response of H9c2 cells stimulated by LPS + ATP, LPS + ATP downregulated the protein expression of PLCγ2, and upregulated the protein expression of PIP2, DAG and IP3. After PE pretreatment, the results were reversed. According to the literatures, P2X7R may also be involved in the activation of the PIP2 signaling pathway. In this experiment, AZD9056 reduced the protein expression of PIP2, DAG, IP3, and upregulate the protein of PLCγ2 (Figure 5B). Meanwhile, immunofluorescence results exhibited that PE and AZD9056 inhibited the fluorescence expression of PIP2 (Figure 5C). In addition, the interaction between PIP2 and P2X7R was enhanced under LPS + ATP stimulation, while the expression of P2X7R binding to PIP2 was reduced after PE treatment (Figure 5E).

Ca^2+^ has an irreplaceable regulatory role in many cellular functions and is a crucial signaling factor in inflammatory responses (Zhou and Tian, 2018). The results of flow cytometry showed that the level of Ca^2+^ in H9c2 cells increased after LPS + ATP induction, but the results were reversed after PE (20 μM) pretreatment. Besides, EGTA is a special calcium chelator, which can inhibit the level of extracellular calcium (Ye et al., 2020). When H9c2 cells were pretreated with EGTA (8 μM), the level of calcium ions was also inhibited, so it could be preliminarily determined that PE inhibited LPS + ATP-induced Ca^2+^ by inhibiting the level of extracellular Ca^2+^ (Figure 5D). In short, P2X7R mediated LPS + ATP-induced activation of PIP2 signaling pathway in H9c2 cells.

### 3.6 PE inhibited the activation of MAPK signaling pathway in LPS + ATP-induced H9c2 cells

**FIGURE 6:**
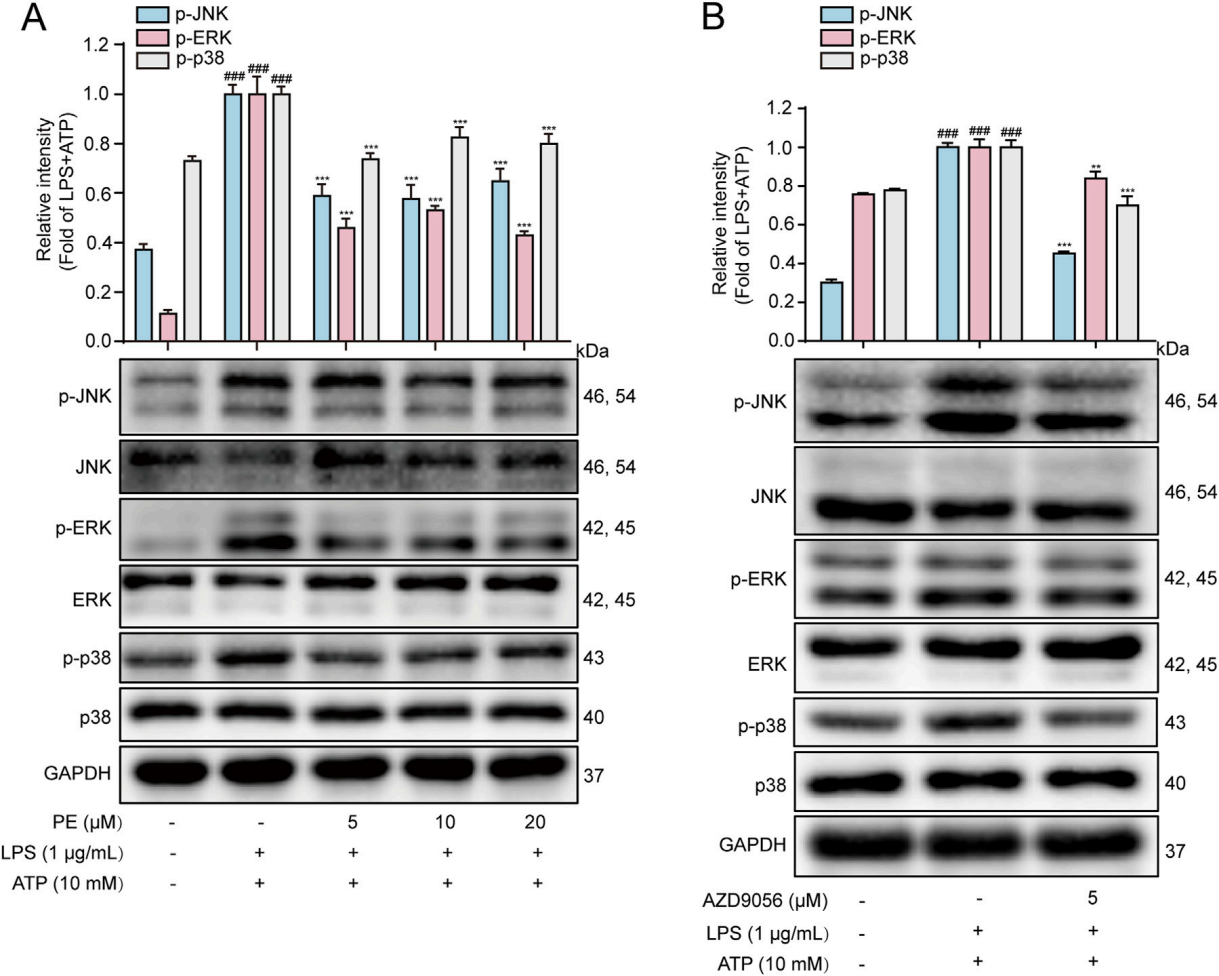
PE inhibited the activation of MAPK signaling pathway in LPS + ATP-induced H9c2 cells. **(A, B)** The protein expressions of p-JNK, JNK, p-ERK, ERK, p-p38 and p38 in H9c2 cells were detected by Western blot (*n* = 3). Compared with control group, ^###^ *P* < 0.001; Compared with LPS + ATP group, ^***^ *P* < 0.001.

MAPK are serine/threonine kinases including p38MAPK, ERK, and JNK that respond to extracellular stimuli and regulate various physiological processes, such as gene expression, stress response, and cell survival or death (Li et al., 2025). LPS and ATP can both cause cellular inflammation by activating the phosphorylation of the above three important MAPK pathway (Zhang et al., 2022). Therefore, MAPK is another important signaling pathway that plays an important role in the inflammatory process (Ren et al., 2020). As shown in Figure 6A, LPS + ATP upregulated the phosphorylation levels of JNK, ERK and P38, while PE pretreatment reversed the protein expression levels. Previous studies have shown that P2X7R may play a role in activating MAPK signaling pathway. In this experiment, AZD9056 reduced the protein levels of p-JNK, p-ERK and p-p38 (Figure 6B). The above results verified that P2X7R is located upstream of MAPK, and PE exerted an inhibitory effect on the MAPK signaling pathway by P2X7R regulation.

### 3.7 PE targeted P2X7R to exert an anti-inflammation effect

**FIGURE 7:**
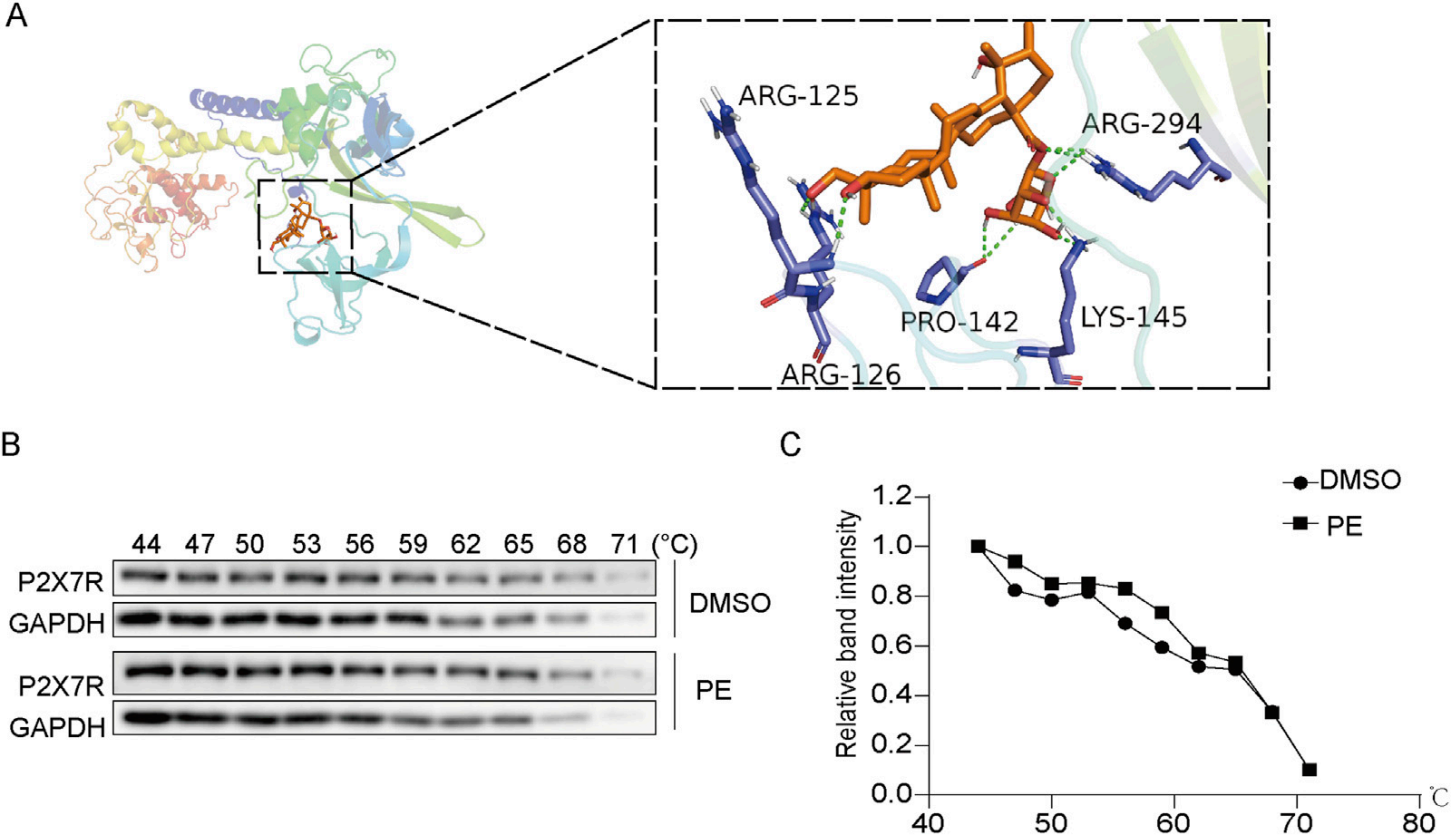
PE targeted P2X7R to exert an anti-inflammation effect. **(A)** The molecular docking of PE and P2X7R; **(B,C)** Cellular thermal shift assay (CETSA) was used to determine the effect of PE on P2X7R protein stability.

As shown in Figure 7A, the molecular docking result showed that PE and P2X7R have multiple binding sites such as ARG-125, ARG-126, ARG-294, PRO-142 and LYS-145, and the binding energy reached −8.0 kJ/mol, which demonstrated the combination of PE and P2X7R. Furthermore, the interaction between PE and P2X7R was explored by molecular docking and CETSA assay. As shown in Figure 7B, P2X7R was degraded in DMSO-treated cells with increasing temperature. However, P2X7R was relatively stable in PE treated cells under the same temperature conditions (Figure 7C). These results indicated that PE could bind to P2X7R and increase its thermal stability.

### 3.8 PE alleviated the cardiac dysfunction in LPS-induced myocarditis rats

**FIGURE 8:**
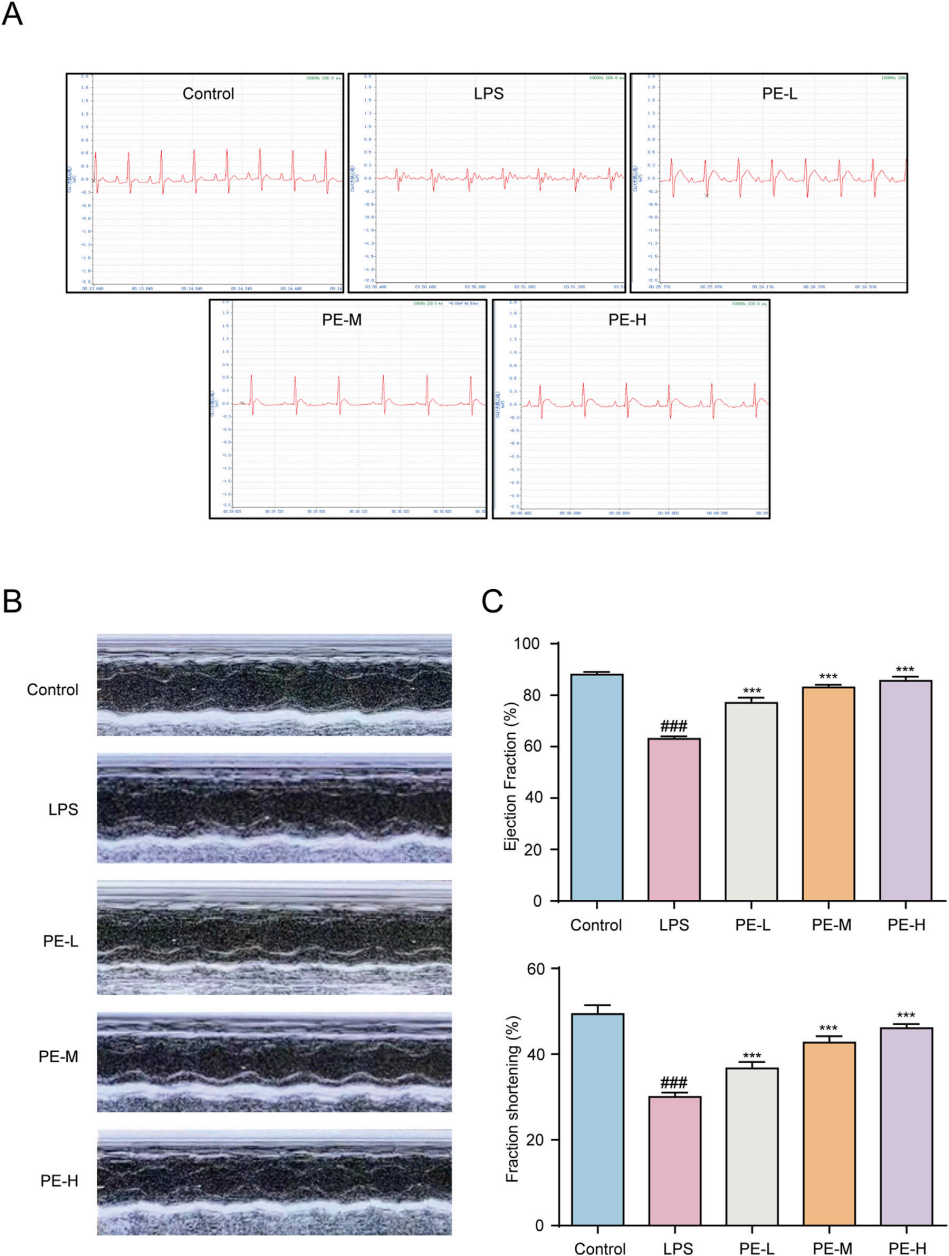
PE alleviated the cardiac dysfunction in LPS-induced myocarditis rats. Rats were injected with PE (10, 20, 40 mg/kg) through tail vein for 5 days, followed by intraperitoneal injection of LPS for 18 h, and anesthetized with 4 mL/kg 10% chloral hydrate. **(A)** ECG of rats was measured; **(B,C)** The echocardiography of rats was measured by MyLab™Six intelligent color ultrasonic diagnostic instrument and counted (*n* = 5). Compared with control group, ^###^ *P* < 0.001; Compared with LPS group, ^***^ *P* < 0.001.

The ECG of the LPS group was abnormal, and the ST segment was elevated, while the PE-L, PE-M and PE-H ECGs recovered significantly, and the ST segment decreased (Figure 8A). The results showed that LPS caused significant abnormalities in the electrocardiogram of rats and might lead to myocardial ischemia, while PE alleviated this injury. EF and FS are often used to indicate and reflect left ventricular systolic function (Li et al., 2019). The EF and FS were reduced in the LPS group obviously, while PE was able to alleviate this situation (Figures 8B,C), suggesting that PE relieved the cardiac dysfunction caused by LPS.

### 3.9 PE ameliorated LPS-induced myocarditis in rats

**FIGURE 9:**
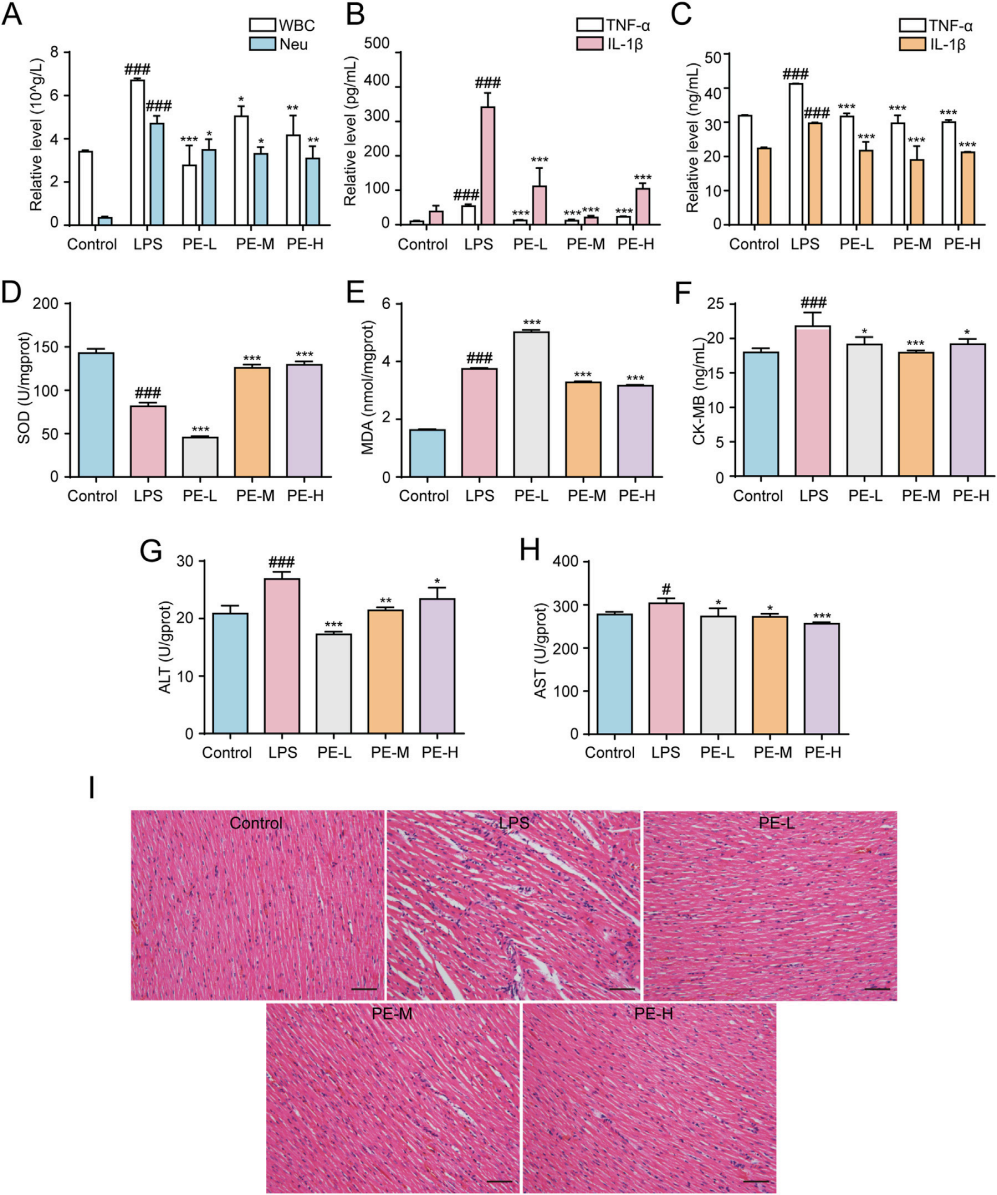
PE ameliorated LPS-induced myocarditis in rats. The rats were injected with PE (10, 20, 40 mg/kg) through tail vein for 5 days, followed by intraperitoneal injection of LPS for 18 h to determine the corresponding blood routine indexes of the rats. **(A)** The number of WBC and Neu in the blood of rats (*n* = 5); **(B)** Release level of TNF-α and IL-1β in serum of rats (*n* = 5); **(C)** TNF-α and IL-1β release level in rat heart tissue (*n* = 5); **(D)** SOD level in rat heart tissue (*n* = 5); **(E)** MDA level in rat heart tissue (*n* = 5); **(F)** CK-MB level in rat heart tissue (*n* = 5); **(G)** ALT level in rat heart tissue (*n* = 5); **(H)** AST level in rat heart tissue (*n* = 5); **(I)** Histopathological evaluation of the heart by H&E staining (200×), scale bar = 100 μm. Compared with control group, ^#^ *P* < 0.05, ^##^ *P* < 0.01, ^###^ *P* < 0.001; Compared with LPS group, ^*^ *P* < 0.05, ^**^ *P* < 0.01, ^***^ *P* < 0.001.

Blood routine analysis revealed that LPS increased the levels of WBC and Neu in the blood of rats, while PE reduced the above index (Figure 9A). As shown in Figures 9B,C, similarly, PE-L, PE-M, and PE-H alleviated the level of TNF-α and IL-1β in the serum and heart tissue increased in LPS group. Additionally, compared with the control group, SOD levels in heart tissue induced by LPS were obiviously decreased, and MDA, CK-MB, ALT and AST levels were significantly increased, while PE effectively ameliorated such damage to a certain extent (Figures 9D–H). Furthermore, H&E staining results displayed the degree of myocardial tissue damage. The cardiomyocytes of the control group were normal without bleeding or neutrophil infiltration, while the myocardial injury in the LPS group was severe, and myocardial fibrosis and inflammatory cell infiltration were seen. PE significantly alleviated this phenomenon, that is, improved myocardial injury in rats (Figure 9I). In a word, PE improved LPS-induced myocarditis in rats.

### 3.10 PE mitigated LPS-induced myocarditis in rats by regulating NLRP3/PIP2/MAPK signaling pathway

**FIGURE 10:**
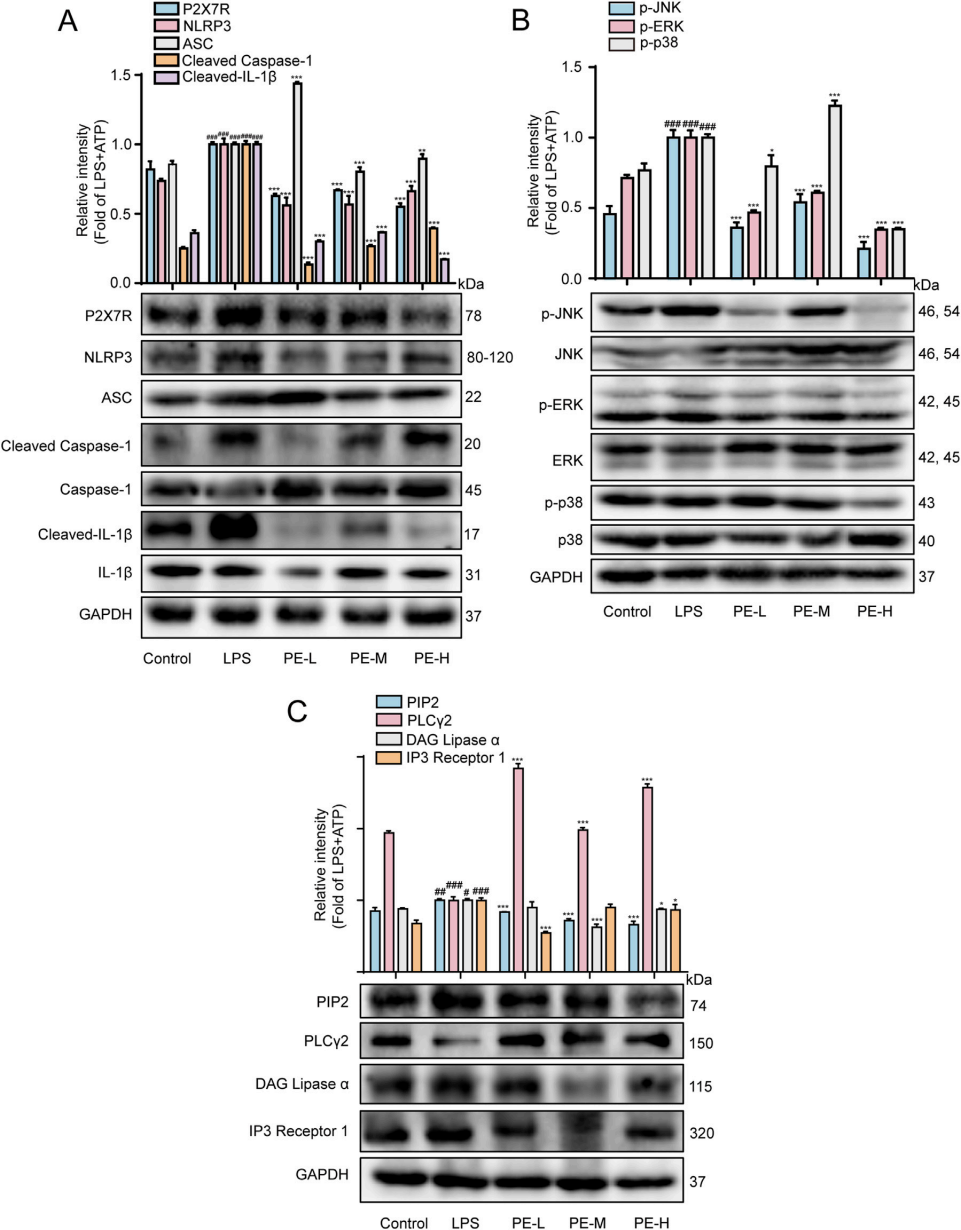
PE mitigated LPS-induced myocarditis in rats by regulating the NLRP3/PIP2/MAPK signaling pathway. The rats were injected with PE (10, 20, 40 mg/kg) through tail vein for 5 days, followed by intraperitoneal injection of LPS for 18 h. **(A)** Western blot was used to detect the protein expression of P2X7R, NLRP3, ASC, Cleaved Caspase-1, Caspase-1, Cleaved-IL-1β, IL-1β in rat heart tissue (*n* = 5); **(B)** p-JNK, JNK, p-ERK, ERK, p-p38, p38 protein expression (*n* = 5); **(C)** Protein expression of PIP2, PLCγ2 and IP3 (*n* = 5). Compared with control group, ^##^ *P* < 0.01, ^###^ *P* < 0.001; Compared with LPS group, ^*^ *P* < 0.05, ^**^ *P* < 0.01, ^***^ *P* < 0.001.

P2X7R produced in the heart tissue, which activated NLRP3 inflammasome after LPS stimulation, with significantly increased expression of P2X7R, NLRP3, ASC, Cleaved Caspase-1 and Cleaved IL-1β in comparison with control group (Figure 10A). LPS upregulated the expression of p-JNK, p-ERK and p-p38, PIP2, DAG, IP3, and downregulated the expression of PLCγ2 (Figures 10B,C). After pretreatment with PE (10, 20, 40 μM), the expression of these proteins was reversed. These data demonstrated that PE inhibited the activation of P2X7R/NLRP3/IL-1β, PIP2 and MAPK signaling pathway, so as to ameliorate myocardial injury in a way.

## 4 Discussion

Traditional Chinese medicine reputes that myocarditis belongs to the category of “palpitations” and “heartache”. The internal cause is caused by the lack of healthy qi, and the external cause is caused by the pathogenic toxin invading the heart including wind-heat pathogenic toxin and damp-heat pathogenic toxin. Therefore, as a medicine that clears heat, detoxifies, promotes *Qi* and cools blood, Jiubiying is suitable for symptoms of myocarditis. According to relevant literature, LPS, a component of the outer membrane of Gram-negative bacteria, serves as an effective inducer of myocarditis. LPS binds to TLR4, activates innate immunity, triggers signaling, and induces pro-inflammatory cytokines, causing myocardial inflammation and injury (Fang et al., 2024). It also causes oxidative stress and mitochondrial dysfunction, further driving myocarditis development (Liu et al., 2023). The effect of the main active compound PE in Jiubiying in improving myocarditis *in vitro* and *in vivo* was deliberated in this study. We prove for the first time that PE could protect from myocarditis through P2X7R/NLRP3/IL-1β, PIP2 and MAPK signaling pathways via binding to P2X7R.

Oxidative stress occurring during inflammation can aggravate the autoimmune process of myocarditis (Lu et al., 2023). Therefore, inhibition of the antioxidant system and long-term oxidative stress may be one of the pathological mechanisms of cardiac remodeling leading to inflammatory cardiomyopathy (Tada and Suzuki, 2016). The results showed that LPS + ATP increased the ROS and MDA levels, and decreased the SOD level and MMP, which was reversed by PE treatment, demonstrating PE has an antioxidant property.

Inflammation is the main cause of further myocardial damage and dysfunction (Chen et al., 2022). Activated neutrophils can secrete cytokines such as TNF-α and IL-1β to further damage the myocardium, leading to metabolic dysfunction, degeneration, and necrosis (Yu et al., 2020). The *in vivo* results showed that PE inhibited the increased numbers of WBC, Neu, and increased levels of TNF-α and IL-1β in LPS-induced myocarditis rats.

A variety of cellular enzymes are stored in the cardiomyocytes, which can reflect the integrity of the cardiomyocytes. When the myocardium is damaged, the cardiomyocytes will experience ischemia or even necrosis, and the permeability of the cell membrane will also change, releasing a large amount of enzyme (Qin et al., 2022). Among them, CK-MB is more stored in cardiomyocytes and less often in tissues other than the myocardium, so it can specifically reflect the extent of myocardial damage (Zhou et al., 2022). In addition, the content of ALT and AST will increase when the tissue is damaged or necrotic. On this basis, the presence of myocardial damage in the body can be judged by detecting its contents (Lazo et al., 2015). The results of this study showed that PE inhibited the elevation of CK-MB, ALT and AST.

The occurrence and progression of myocarditis is inseparable from the mediation and participation of NLRP3 inflammasome (Toldo and Abbate, 2024). It is a good choice to inhibit the activation of NLRP3 inflammasome and downstream inflammatory pathways to improve cardiac function of myocarditis rats. In the inflammatory response, P2X7R performs crucial function. LPS stimulated P2X7R synthesis and inflammasome assembly, inflammasome production and further IL-1β release (Rowell et al., 2020). Experimental data made it clear that PE had a certain inhibitory effect on the activation of P2X7R and NLRP3 inflammasomes, and AZD9056 (P2X7R inhibitor) also obviously inhibited the activation of NLRP3 inflammasomes. Furthermore, the molecular docking and CETSA results both showed that PE and P2X7R have a good combination. Therefore, it was determined that PE improved myocardial injury and LPS + ATP-induced H9c2 cell damage by inhibiting P2X7R-mediated NLRP3-dependent IL-1β secretion via binding to P2X7R.

PLC activation can mediate the cleavage of PIP2 into DAG and IP3, thereby activating Ca^2+^ (Bang et al., 2021). PE inhibited the release of extracellular Ca^2+^, which might be affected by the PIP2 signaling pathway. The amino acid sequence of the proximal C-terminal region of the P2X receptor revealed the presence of two clusters of basic residues that form the regulatory PIPn binding site in most subunits (Xiao et al., 2018). However, there are few reports on the activation of PLC by P2X7, and K^+^ depletion has been proposed as a mechanism. Furthermore, the regulation of downstream effects of PLC by P2X7 has been reported. In microglia, P2X7-induced Ca^2+^ elevation was found to increase DAG lipase activity, which is produced by PLC (Lu et al., 2024). And negative modulation of P2X7 via consumption of PIP2 has also been reported (Ma et al., 2022). So far, no direct interaction between PIP2-P2X7R has been found. Nevertheless, the Co-IP results well proved that the interaction between PIP2 and P2X7R was enhanced under LPS + ATP stimulation, and the binding between P2X7R and PIP2 were decreased after PE treatment. Besides, in this experiment, it was found that when P2X7R was inhibited with its inhibitor AZD9056, the expressions of PIP2, DAG, and IP3 were also inhibited accordingly, indicating that when H9c2 cells were induced with LPS + ATP, P2X7R participated in the activation of PIP2 signaling pathway, but the specific connection between them needs to be further ascertained.

Three members of MAPKs are phosphorylated upon P2X7R activation, namely, the closely related extracellular signal-regulated kinases ERK1 and ERK2, JNK and p38 MAPK, activated by stimuli including inflammatory signals and stress, and these kinases are involved in inflammation, apoptosis and proliferation. The results showed that PE improved myocardial injury by inhibiting the MAPK signaling pathway and P2X7R was also involved in the activation of MAPK signaling pathways. But the relationship between them needs further investigation.

Overall, PE improved LPS + ATP-induced H9c2 cell injury and had a protective effect on LPS-induced myocarditis rat. In addition, it’s found for the first time that P2X7/NLRP3/IL-1β, PIP2, and MAPK signaling pathways are involved in the regulatory effect of PE on myocarditis, providing a prospect for development and application of myocarditis drugs.

## 5 Conclusion

In this study, we investigated the protective effects and mechanism of PE on LPS-induced myocarditis. The results illustrated that PE showed a markedly anti-myocarditis activity *in vitro* and *in vivo*. Mechanistically, PE could bind to P2X7R and inhibit the binding between P2X7R and PIP2, subsequently inhibited the activation of NLRP3 inflammasome, PIP2 and MAPK signaling pathways. Therefore, PE may be a promising drug for the treatment of myocarditis.

## Data Availability

The raw data supporting the conclusions of this article will be made available by the authors, without undue reservation.

## Abbreviations

CK-MB, creatine kinase-MB; Co-IP, co-immunoprecipitation; DAG, diacylglycerol; DCFH2-DA, 2′, 7′-Dichlorodihydrofluorescein diacetate; DMEM, Dulbecco’s modified eagle medium, ECG, Electrocardiograph; EF, ejection fraction; EGTA, ethylene glycol tetra acetic acid; ELISA, Enzyme-linked immunosorbent assay; FBS, fetal bovine serum; FS, fractional shortening; H&E, hematoxylin-eosin; IP3, inositol 1, 4, 5-trisphosphate; LDH, lactate dehydrogenase; MAPK, mitogen-activated protein kinase; MDA, malondialdehyde; MMP, mitochondrial membrane potential; MTT, (4, 5-Dimethylthiazol-2-yl)-2, 5-diphenyltetrazolium bromide; Neu, neutrophils; NLRP3, nod-like receptor 3; PE, Pedunculoside; PIP2, phosphatidylinositol 4, 5-bisphosphate; PVDF, polyvinylidene fluoride; P2X7R, P2X7 receptor; ROS, reactive oxygen species; SOD, superoxide dismutase; TNF-α, tumor necrosis factor-α; WBC, white blood cells.
