# Supplementary figures and images for: Pedunculoside targets P2X7R to protect against myocarditis by regulating the NLRP3/PIP2/MAPK signaling pathway
Source: Front Pharmacol. 2025 Jun 26;16:1589298. doi: 10.3389/fphar.2025.1589298 (PMC12240787; doi:10.3389/fphar.2025.1589298)

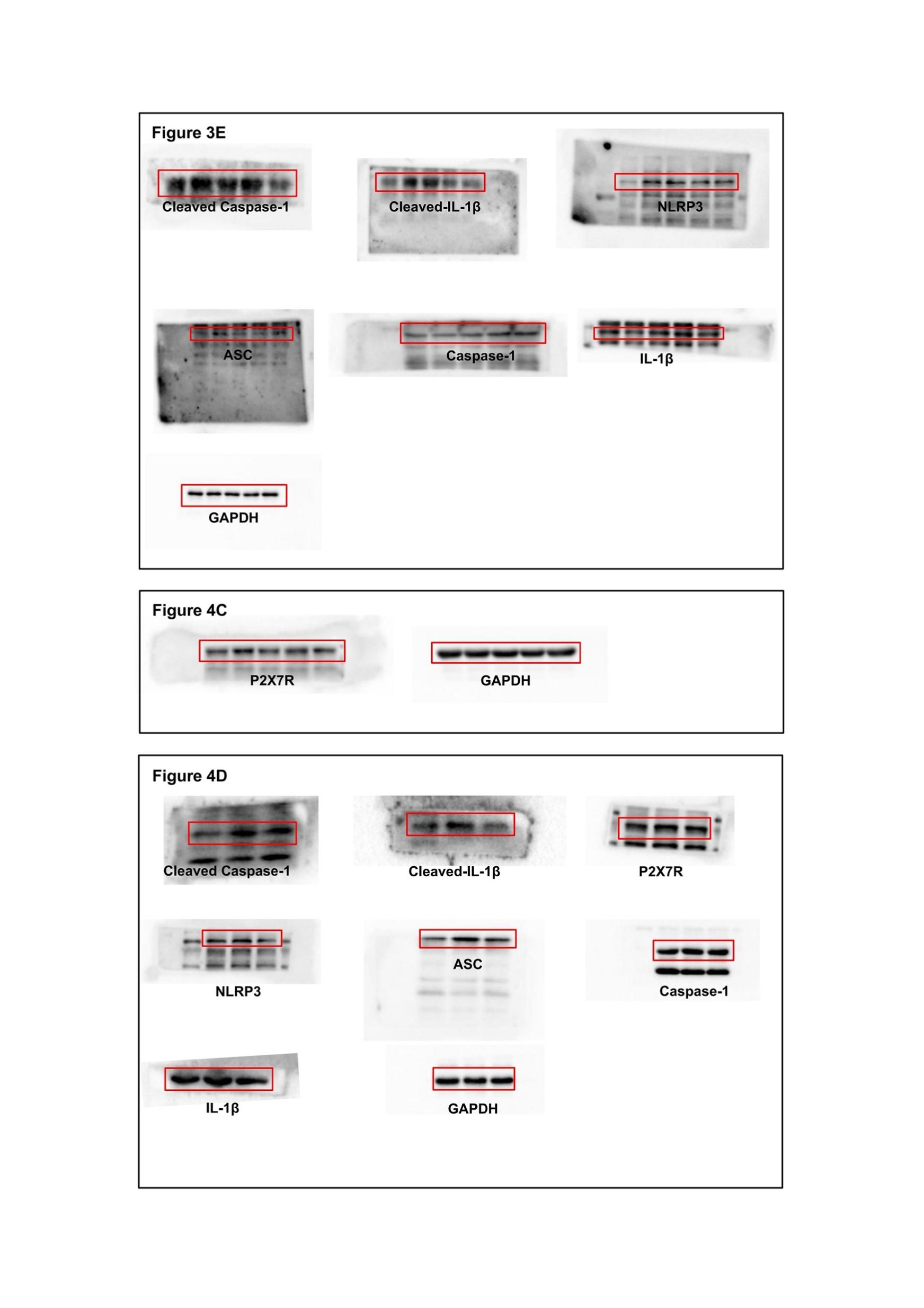


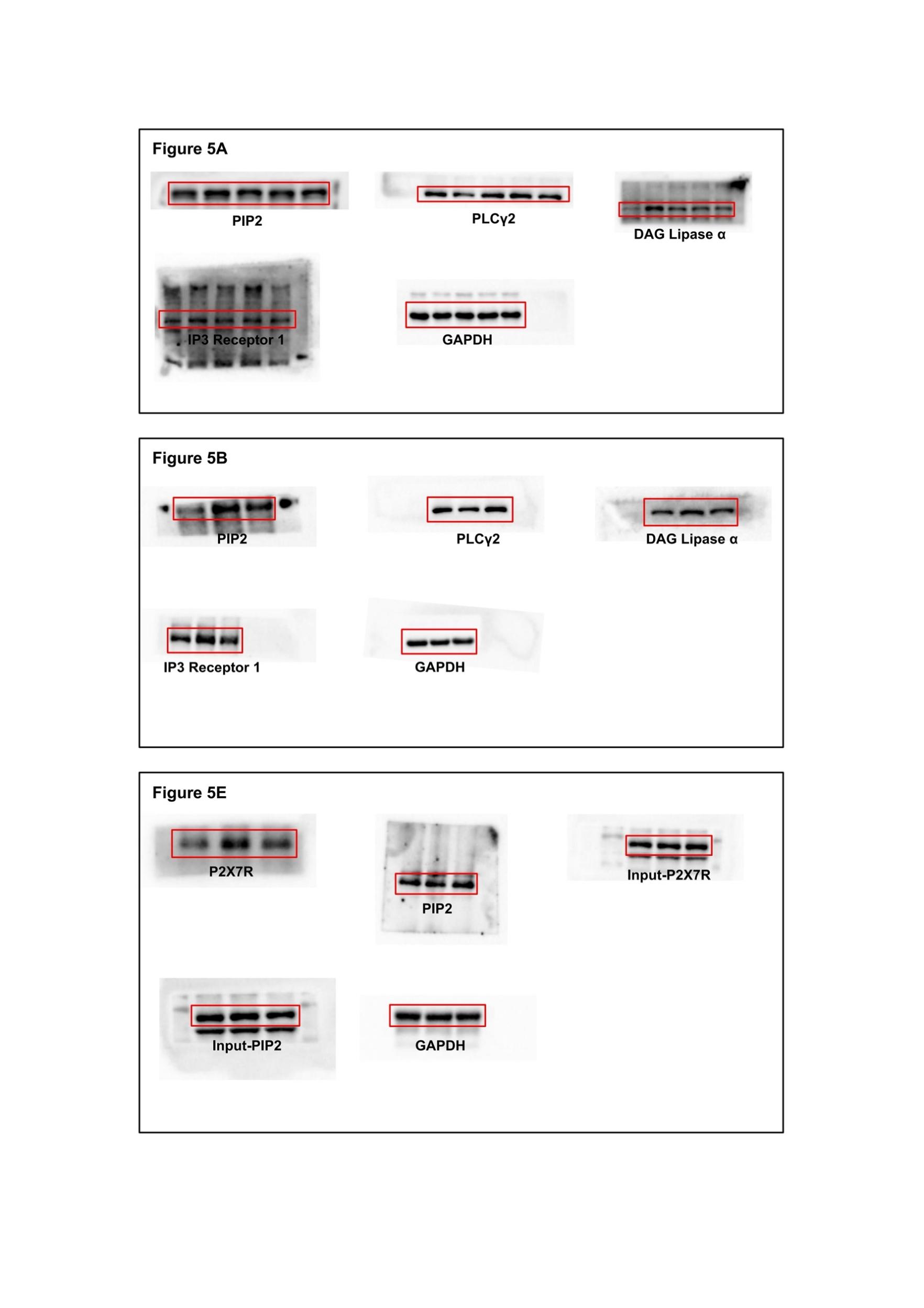


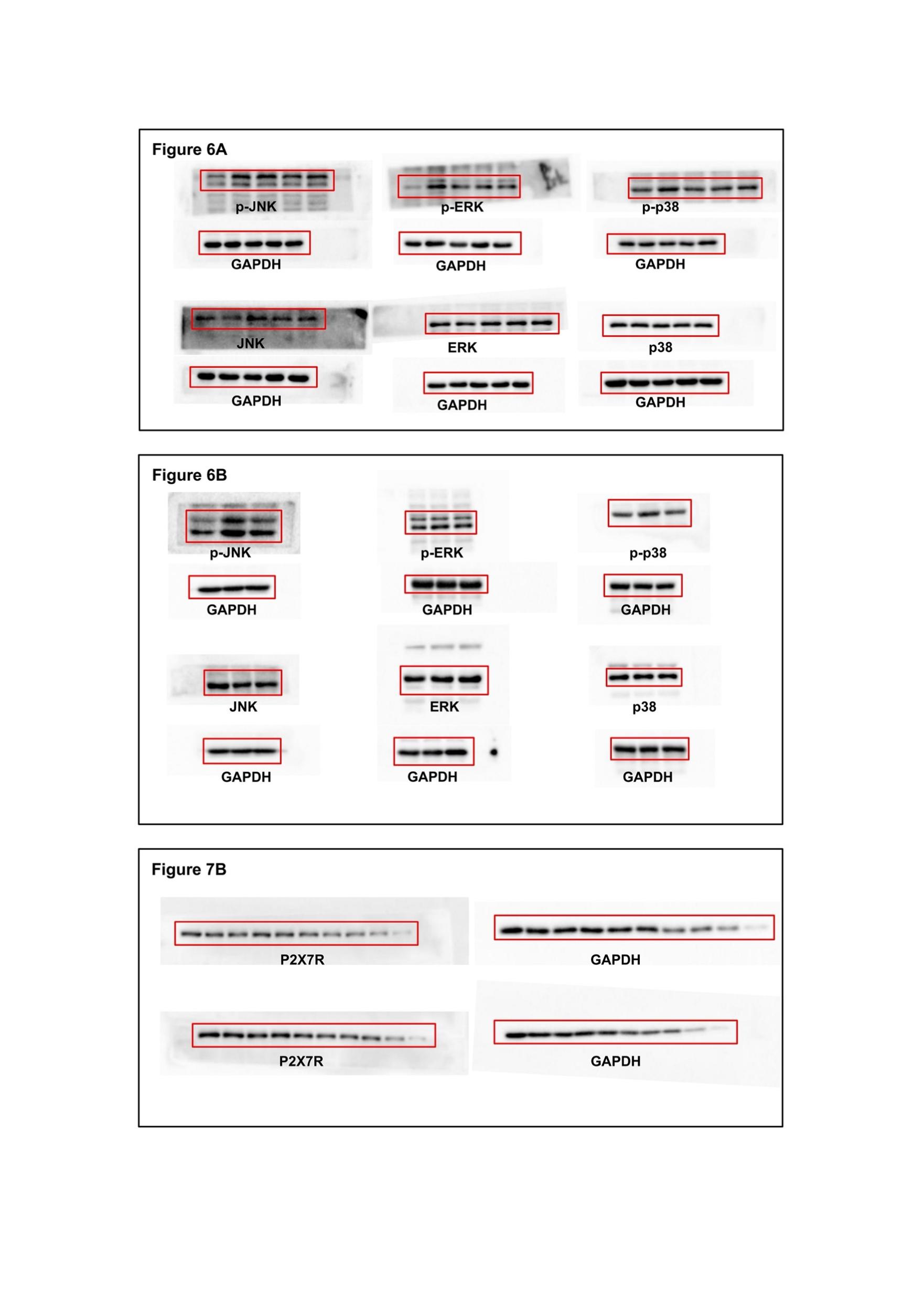


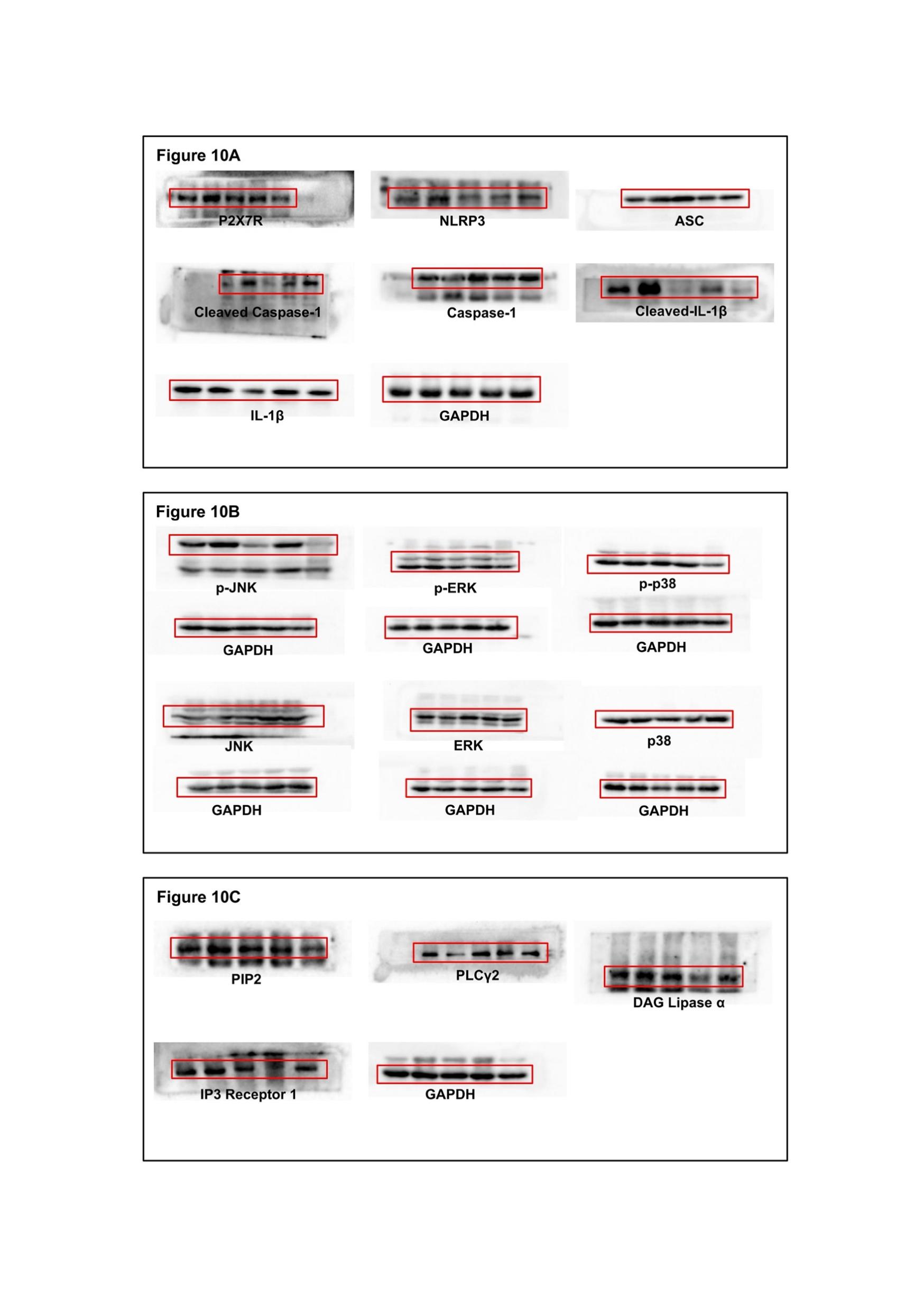

Supplement: Supplementary file 1 [file Supplementaryfile1.docx]
